# Impact of visual and textural characteristics of street walls on stress recovery

**DOI:** 10.1038/s41598-024-64618-z

**Published:** 2024-07-02

**Authors:** Nan Zhang, Lin Zhao, Jin Shi, Weijun Gao

**Affiliations:** 1 Innovation Institute for Sustainable Maritime Architecture Research and Technology, Qingdao, 266033 China; 2 Faculty of Environmental Engineering, The University of Kitakyushu, Kitakyushu, 8080133 Japan; 3 College of Architecture and Urban Planning, Qingdao University of Technology, Qingdao, 266033 China

**Keywords:** Visual and textural characteristic, Street wall, Stress recovery, HRV, Psychophysiological health, Environmental social sciences, Health care

## Abstract

Rapid urbanization increases psychological stress among pedestrians, potentially heightening mental health disorders. This study examines the role of street walls' visual and textural characteristics in stress recovery, using Qingdao as a case study. Virtual reality is employed to simulate five distinct street walls: yellow mortar, brown stone, red brick, green plant, and white mortar. The stress recovery effectiveness of these walls was evaluated through psychological and physiological indicators from 48 young college students. Results indicated that street walls with warm tones, particularly brown stone, significantly aid stress recovery. Psychologically, Restorative Components Scale was highest for brown stone at 1.13. Physiologically, it was linked with notable reductions in diastolic and pulse pressure (decreases of 2.95 mmHg and 2.27 mmHg, respectively), and enhanced parasympathetic activity, as evidenced by the fastest decrease in low frequency/high frequency ratio (LF/HF), and increases in pNN50 and RR (0.14–2.01% and 1.57–11.81 ms, respectively). For urban design, the incorporation of warm-toned materials and natural elements like stone is recommended for their superior restorative benefits.

## Introduction

The contemporary trends of globalization and urbanization have ushered in heightened levels of traffic noise, pollution, and mental strain, correlated with escalating instances of mental disorders^1,2^. Extensive research underscores the restorative potential of natural settings like lakes and forests, bolstering both physical and mental well-being^3–5^. Elsadek et al. highlighted the psychophysiological benefits of plant interactions for stress recovery^6^, and Finlay et al. underscored the positive impacts of blue-green spaces on public health^7^. However, limited access and exposure to these natural settings constrain their benefits for health promotion. Consequently, scholars are increasingly focusing on the restorative advantages of urban environments^8,9^. A well-designed urban environment can enhance subjective perceptions, alleviate mental fatigue, and increase happiness among residents^9,10,11^. Since streets are the urban spaces most frequently encountered by residents, enhancing their restorative qualities is essential^12^.

While existing inquiries concentrate predominantly on street vegetation^13,14^, Lohr et al. have established the calming effects of trees, rendering tree-lined streets more appealing^15^. Similarly, Lindal et al. have shown that floral elements significantly enhance street restorativeness^16^. Other research has explored the impact of architectural complexity, traffic volume, and pedestrian flow on street restorability^17–19^. However, the influence of the street interface itself, particularly street walls, on stress recovery is less studied. Asgarzadeh et al. noted that taller building fronts along streets might heighten oppression and stress^20^, while Cui et al. highlighted the significant role of building materials^21^. Despite the focus on building interfaces, the walls that form part of these interfaces have been somewhat overlooked. In Qingdao’s historic district, distinct from the modern high-rise facades, various walls typify the streetscape. During the German lease period, urban planning led to the predominance of low-rise, German-style garden houses with walls of stone and predominantly yellow tones separating private gardens from the streets^22^. Over time, these evolved to include brick constructions and, later, white mortar plastering with wrought iron railings during renovations^23^. Historical elements are shown to positively influence stress recovery, with Van et al. finding that historical streets aid more in fatigue recovery than alleys and modern commercial streets^24^. Roe et al. also observed that historic streets enhance positive emotions in residents^25^, contributing to both stress recovery and well-being^26^. Positive perceptions can enhance well-being by fostering feelings of satisfaction and contentment, while negative perceptions can lead to stress and diminished health^24–26^. Considering Qingdao’s reputation as a livable city, with up to 76% of its area deemed suitable for living^27^, it is imperative during periods of urban development and transformation that the government protects beneficial historical elements and ameliorates detrimental ones like inappropriate colors to foster a healthy, sustainable urban environment^28^. The preservation and promotion of historical street walls, proven to facilitate stress recovery, should be a focal point in urban renewal strategies. Lyu et al. emphasized the importance of walls in enhancing the quality of spatial perception and resident happiness in Qingdao^29^. Therefore, examining the effects of street walls with varying visual and textural characteristics on stress recovery is essential.

The combination of subjective evaluations and physiological indices is a standard methodology for assessing stress recovery^30,31^. Building upon the Attention Recovery Theory (ART) developed by Rachel and Stephen^32^, the Restorative Components Scale (RCS) evaluates stress recovery through four dimensions: being away, extent, fascination, and compatibility, with its effectiveness demonstrated by Laumann et al. and Yin et al.^33,34^. Physiologically, stress recovery correlates significantly with cardiovascular system activity, with blood pressure (BP) and heart rate variability (HRV) serving as reliable indicators. Exposure to environments rich in natural elements has been shown to significantly lower systolic and pulse pressures and reduce stress levels^35,36^. HRV parameters, indicative of autonomic nervous system (ANS) activity, reveal that a predominance of parasympathetic nervous system (PNS) activity corresponds to reduced stress levels, evidenced by decreased low frequency/high frequency ratio (LF/HF) and increased pNN50 and RR^37–39^. Moreover, virtual reality (VR) technology offers a controlled, immersive environment for studying human-environment interactions, minimizing external disturbances like noise and weather^40^. The alignment between virtual reality and field observations has been substantiated by Xiang et al.^41^, establishing VR as a viable method for recreating street environments for research purposes.

In this study, virtual reality (VR) technology was utilized to simulate different street walls. The effectiveness of these walls in facilitating stress recovery was evaluated using two primary methods: psychological indicators via RCS questionnaire, and physiological indicators—blood pressure (systolic, diastolic, and pulse pressure) and HRV parameters (LF/HF, pNN50, RR). The aim of this study is to establish a quantitative method for evaluating the stress recovery potential of street walls and to offer recommendations for optimizing the visual and textural characteristics of walls in historic districts.

## Materials and methods

In this study, 48 young college students were exposed to street walls with five distinct visual and textural characteristics using an HTC Vive Pro device. Their psychological and physiological responses were recorded after stress stimuli. One-way analysis of variance (ANOVA) was employed to analyze the differences between the groups.

### Participants

In June 2022, a recruitment notice was posted at the College of Architecture and Urban Planning, Qingdao University of Technology, seeking participants who were in good health and had no history of mental illness or cardiovascular disease. The sample size was determined using G*Power, considering a study design with within-group analysis and one-way ANOVA. To ensure meaningful results, studies generally require a statistical power over 80% (1 − β = 0.8) and a significance level of *p* < 0.05. Assuming (1 − β)¼ = 0.8, α¼ = 0.05, with an expected effect size of 0.25 and a sample size of 16^42^, a total of 48 participants were recruited to account for potential data contamination. The cohort consisted of 22 men and 26 women, all of whom were either undergraduate or postgraduate students in architecture and residents of Qingdao. Table 1 presents their basic information. Participants were fully briefed on the experimental procedures, associated risks, and confidentiality policies, and provided signed informed consent prior to participation. To minimize variations in physiological indicators, participants were instructed to abstain from consuming alcohol or caffeinated products for at least 12 hours and avoid strenuous exercise for 2 h before the experiment^42,43^. This study was approved by Ethics Committee of Qingdao University of Technology and it have been performed in accordance with the Declaration of Helsinki.

**Table 1:** Participants’ characteristics (n = 48).

Gender	Mean (SD)	Range
Males		
Age	18.67 ± 1.16	18–20
Height (cm)	173.33 ± 2.89	170–175
Weight (kg)	64.33 ± 3.79	60–67
BMI (kg/m^2^)	21.23 ± 5.14	19.56–22.69
Females		
Age	24 ± 1.35	22–27
Height (cm)	164.00 ± 6.63	156–173
Weight (kg)	53.22 ± 6.01	48–65
BMI (kg/m^2^)	21.16 ± 3.16	20.04–22.13

## Environments

### Equipment

Utilizing a Head Mounted Display (HMD), specifically the HTC Vive Pro, participants were immersed in simulated environments. The HTC Vive Pro HMD boasts a combined resolution of 2880 × 1660 pixels, operates at a refresh rate of 90 Hz, and encompasses a field of view angle spanning 110°. During the experimental sessions, participants were outfitted with a blood pressure monitor (Yuwell YE660) and an ECG device (Healink-R211B). Table 2 shows the experimental equipment configuration.

**Table 2:** Experiment equipment.

Type	Test program	Range	Accuracy/Sampling rate
HTC Vive Pro	Virtual scene	110°	2880 × 1660
Yuwell YE660	Blood pressure	0–299 mmHg	± 4 mmHg
Healink-R211B	Heart rate Variability	0.6–40 Hz	1000 Hz

### VR environments

**Figure 1:**
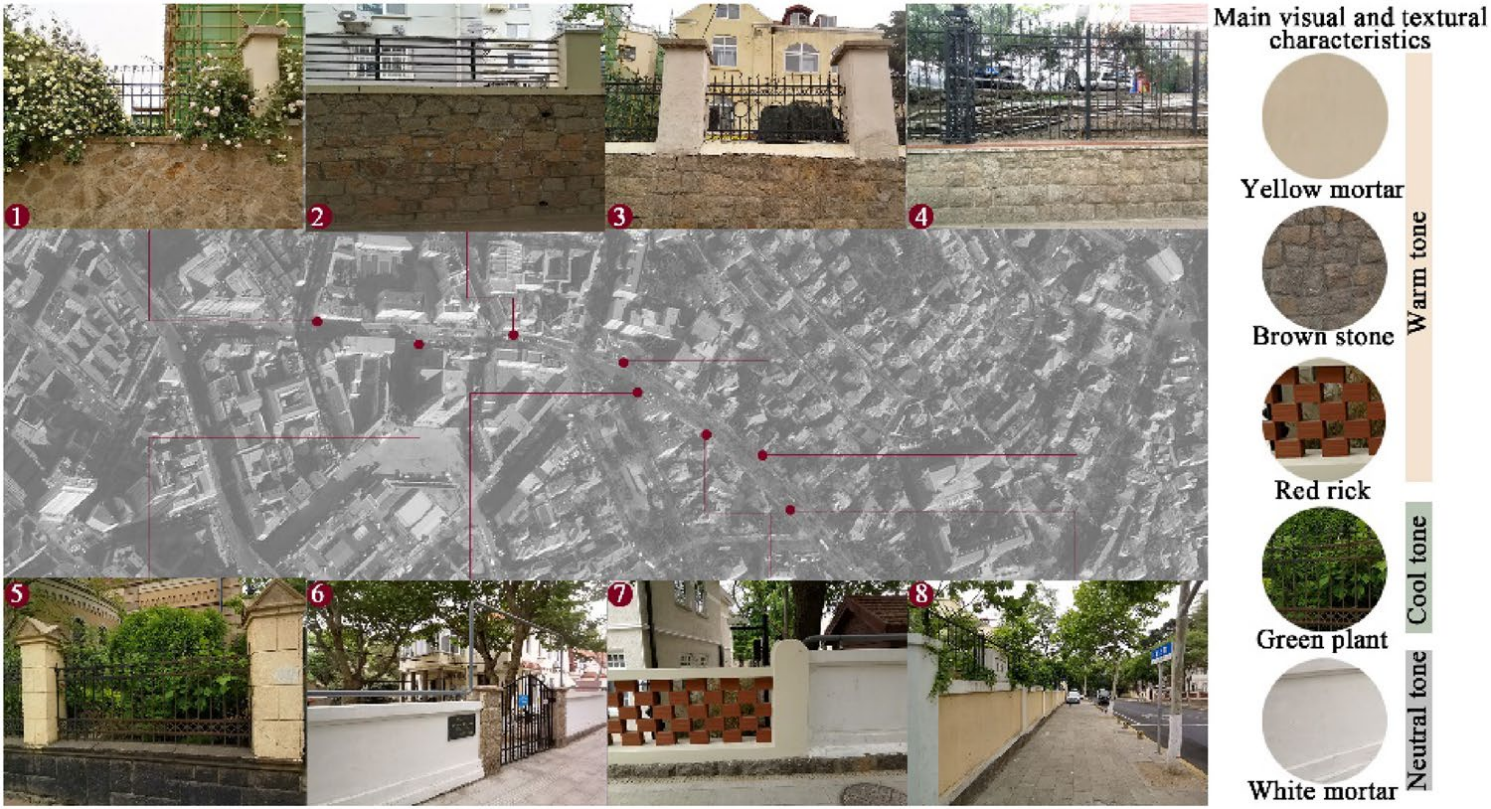
Types of street wall on Dexian Road.

A basic survey was conducted across historical districts in Qingdao, revealing that rubble and brick are the main materials used, predominantly in yellow, white, and red colors. It was observed that some walls equipped with wrought iron railings were densely covered with vegetation. Located in the historical district of Dabaodao, Dexian Road is identified as one of the earliest streets in Qingdao. It served as the dividing line between the residences of foreigners and Chinese during the German lease period, embodying the typical characteristics of the built environment in Qingdao's historical districts. This street holds distinct significance in the history of Qingdao's urban development^23^. Dexian Road is used as an example to illustrate the types of street walls, as shown in Fig. 1.

**Figure 2:**
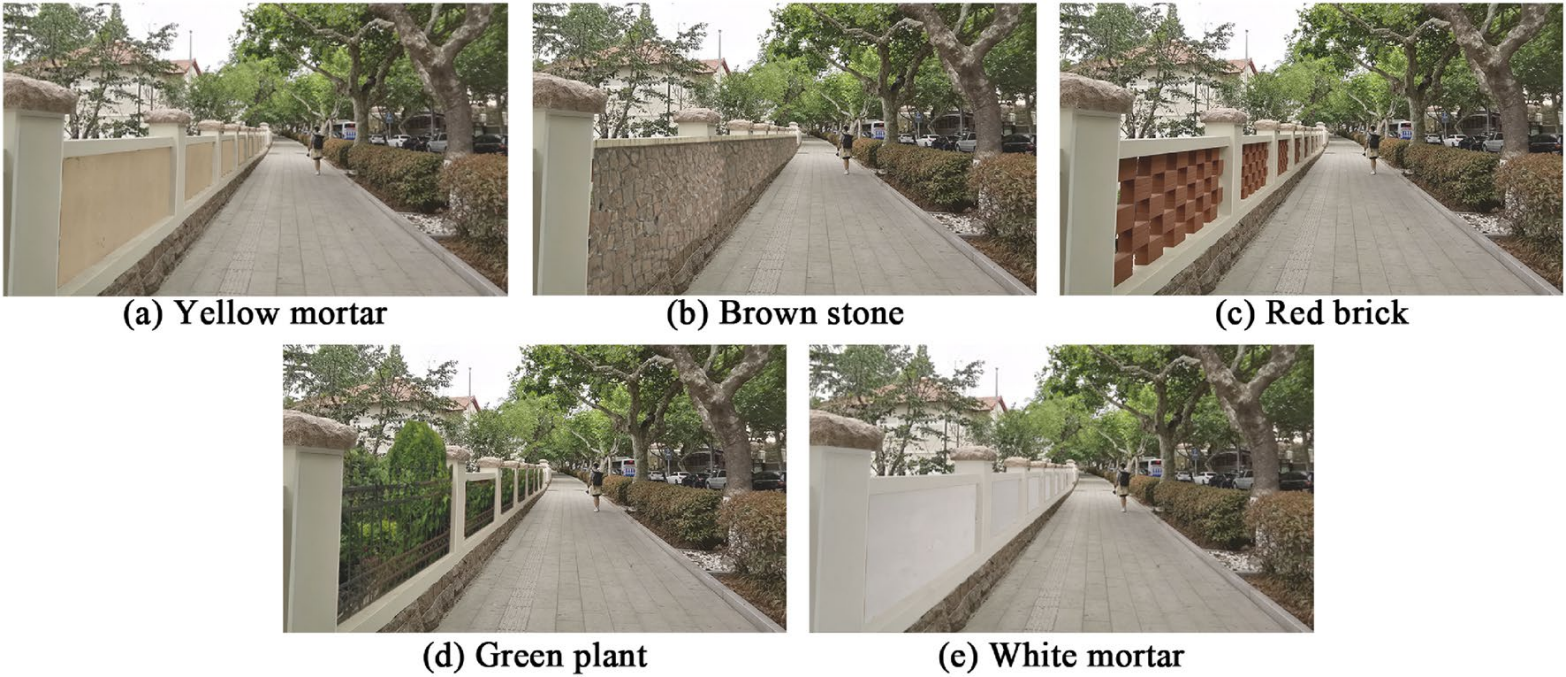
VR scenes of different street walls.

The wall style represents a complex system of material, texture, color, and structural layers. To economize on time and costs, the experiment compared five typical visual and textural characteristics: (a) yellow mortar, (b) brown stone, (c) red brick, (d) green plant, and (e) white mortar. Photoshop 2022 was employed to standardize the visual characteristics of other street elements, ensuring that only the wall features differed, as shown in Fig. 2, which displays the viewing screen in the VR equipment. To focus participants’ attention on the wall and minimize distractions caused by the potential to view the 360° street scene, the study utilized 2-dimensional photos with a fixed field of view^44^. The photos, taken from real scenes on Dexian Road, were shot at a height of 1.75m, matching the average adult eye-level angle^17^. Care was taken to align the center of each photo with the midpoint of the VR device screen to prevent skewing or distortion. Preliminary experiments indicated that VR image stimulation was sufficient to evoke psychophysiological responses. Participant interviews post-experiment confirmed that the VR experience closely mirrored real-world perceptions.

### Procedures

**Figure 3:**
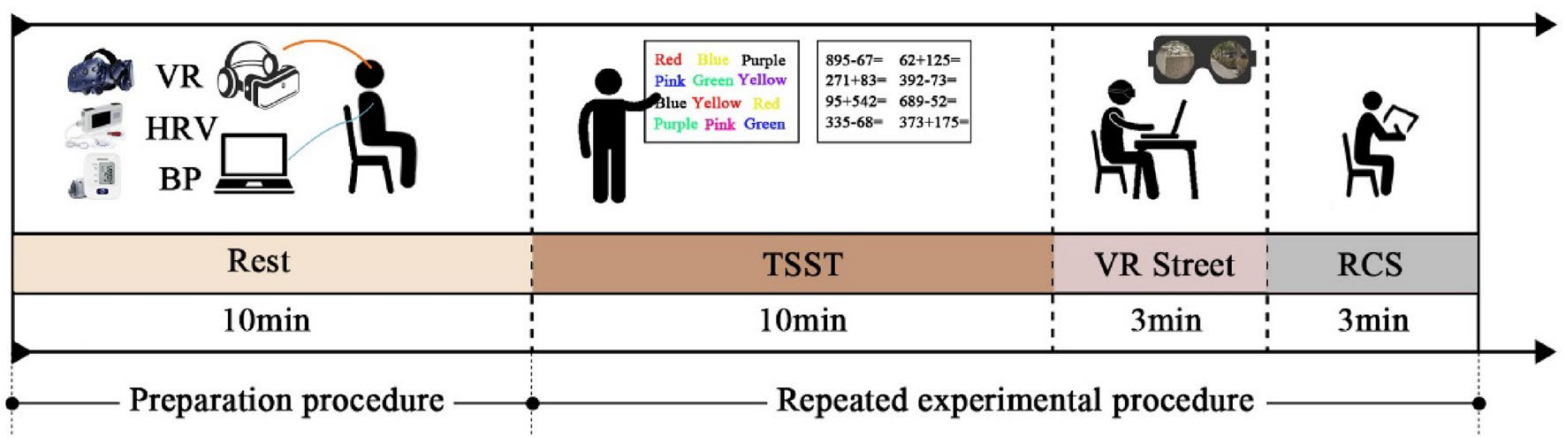
Experiment procedures.

The experimental timeline encompasses the period from July 4th to July 31st, 2022, occurring within the interval of 8:00–12:30 AM. A cohort of 48 participants un-derwent randomized test sequencing, with each participant engaging in the protocol individually. Figure 3 shows the experiment procedures. In the primary stage, participants donned physiological monitoring apparatus (BP and HRV), observing a 10-minute quiescent interval to mitigate initial activity-related confounds. Subsequently, the Trier Social Stress Test (TSST) was performed. The TSST is frequently employed to induce stress in participants and is considered more reliable than stress elicited through noise exposure, emotional induction, cognitive tasks alone, or public speaking alone^45,46^. Comprising two tasks—swift color-word congruence assessment and mental arithmetic computations constrained within a 1000-unit threshold—the TSST spanned 10 minutes. Proceeding this, the third stage ensued, involving participants' interaction with VR equipment to perceive a randomly selected experimental scenario for a duration of 3 min. Throughout this phase, continual HRV data monitoring was upheld, concurrently with blood pressure measurements taken at both commencement and culmination. The fourth stage necessitated participants' completion of the RCS questionnaire post-observation. The experimental procedure was subsequently reiterated, employing diverse scenes, their assortment and order adhering to randomization principles.

### Psychological indicators

The assessment of subjective stress recovery was accomplished using the RCS. Rooted in the Perceptual Restorative Scale (PRS), a widely employed tool for gauging environmental revitalization benefits, RCS presents a more pertinent measurement structure aligned with the four environmental dimensions delineated by ART: being away, extent, fascination, and compatibility^33^. Previous inquiries have substantiated its efficacy in exploring anticipations and prevailing levels of street recovery^34^. As delineated in Table 3, RCS encompassed 15 items, with participants assigning ratings on a seven-point Likert scale, ranging from -3 (no perception) to 3 (strong perception). For the first item of RCS, participants rated their sense of freedom from work and schedule constraints on a seven-point Likert scale: -3 representing 'Totally disagree, I feel completely unfree and still tied to my work and schedule,' -2 for 'Mostly disagree, I don't feel free most of the time,' − 1 indicating 'Slightly disagree, I don't feel free sometimes,' 0 as 'Neutral, with no discernible sense of feeling free or not,' + 1 for 'Slightly agree, I sometimes feel more free,' + 2 as 'Mostly agree, I feel more free most of the time,' and + 3 representing 'Totally agree, I feel very free, completely free from the constraints of work and schedule.' The scoring rules were explained to participants before the experiment commenced.

**Table 3:** Restorative components scale.

ART	RCS Statement
Being away	When I am here, I feel free from my work and regular schedule
	When I am here, I feel free from other people’s requirements and desires
	When I am here, I do not need to consider my duties or responsibilities
Extent	The elements here work together
	The existing elements are appropriate here
	The surroundings make sense
Fascination	There is a lot to discover here
	This setting has numerous things that I wonder about
	There are many items here that attract my attention
	There is plenty that I want to linger on here
	I am engrossed in these surroundings
Compatibility	The surroundings give me the opportunity to do activities that I like
	I can handle the kinds of issues that arise here
	I can rapidly adapt to this environment
	There is an accordance between what I enjoy doing and this environment

### Physiological indicators

#### Blood pressure (BP)

The arterial blood pressure parameter functions as a marker of cardiovascular system functionality, wherein systolic blood pressure (SBP) and diastolic blood pressure (DBP) exhibit correlations with pressure levels. Acknowledging the temporal dynamics influencing SBP and DBP, and taking into account endorsements from select studies that emphasize the relevance of pulse blood pressure (PBP) as a predictive factor for health outcomes, this parameter is also encompassed. PBP, denoting the discrepancy between SBP and DBP, is introduced to enrich the analytical perspective^47^.

#### Heart rate variability (HRV)

HRV parameters offer insights into an individual's stress levels by gauging the interplay between sympathetic nervous system (SNS) and parasympathetic nervous system (PNS) activity^48,49^. Employing the ultralight portable ECG dynamic monitor (Heallink, China), we acquired continuous single-channel ECG data at a 1000 Hz sampling frequency. Data processing was performed using the Kubios HRV Premium software. As delineated in Table 4, three HRV parameters—LF/HF^50,51^, pNN50^49^, and RR^52^—were chosen to appraise participants' stress recovery.

**Table 4:** HRV indicator definition.

Index	Definition	Relationship
LF/HF	The frequency domain parameters consist of low frequency (LF; 0.04–0.15 Hz) and high frequency (HF; 0.15–0.40 Hz)	A higher LF/HF indicates stronger SNS activity and a more aroused state
pNN50	The percentage of successive heartbeat intervals exceeding 50 ms	A higher pNN50 corresponds to increased PNS activity and a more relaxed physiological state
RR	Consecutive normal R-R intervals	A longer RR interval indicates greater relaxation of PNS

### Statistical analysis

For a more comprehensive comprehension of the differential influence of various street wall compositions on both physical and mental recovery, statistical analysis was executed employing SPSS 27.0 software. Pearson correlation analysis was undertaken on the acquired psychological and physiological data. The correlation strength was denoted by the correlation coefficient "r," which spans from − 1 to 1. Positive values signify positive correlations, while negative values denote negative correlations. Generally, upon taking the absolute value, the results are interpreted within five tiers: (i) 0–0.09: negligible correlation; (ii) 0.10–0.39: weak correlation; (iii) 0.40–0.69: moderate correlation; (iv) 0.70–0.89: strong correlation; (v) exceeding 0.90: very strong correlation^53^. Furthermore, to scrutinize variations amidst different aspect ratios, one-way analysis of variance (ANOVA) was applied. The significance levels of correlation and one-way ANOVA are typically conveyed through p-values, categorized into three strata: (1) *p* < 0.001, indicating a highly significant difference; (2) *p* < 0.01, reflecting a significant difference; (3) *p* < 0.05, indicative of a weakly significant difference.

## Results

ANOVA conducted on BP, LF/HF, pNN50, and RR following the TSST stimulation stage revealed no significant differences among the five visual stages of stimulation (*p* > 0.05). This outcome suggests that participants did not develop adaptive responses to the TSST, indicating that their stress recovery was solely influenced by the visual stimulation of the street wall, rather than variations in stress levels induced by the TSST.

### Influence of different street walls on physiological responses

**Figure 4:**
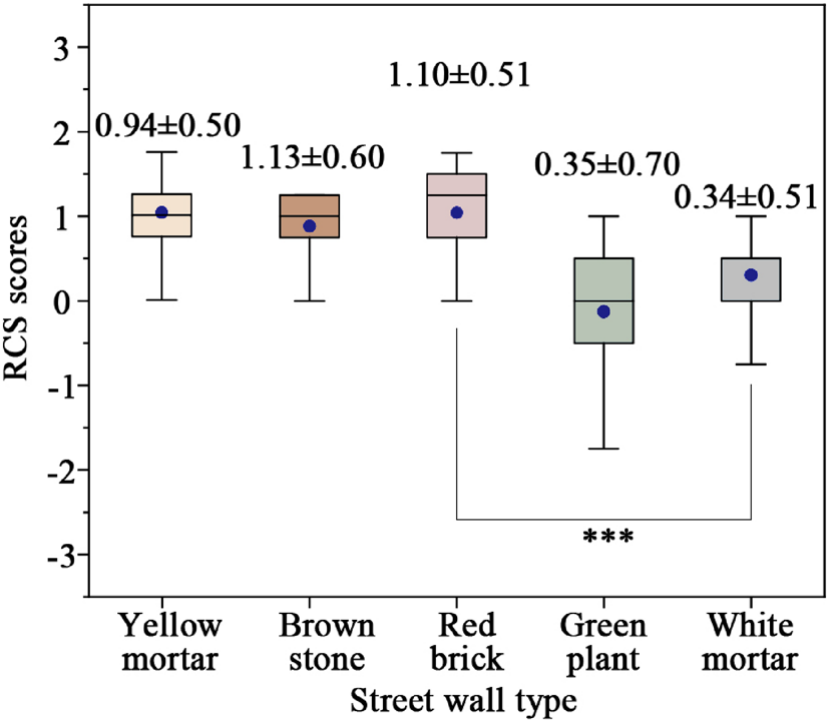
RCS scores for different street walls.

Figure 4 shows RCS scores for different street walls. Notably, all five street wall compositions exhibit discernible stress recovery attributes, each yielding restorative perceptual evaluations surpassing 0. Among these, the brown stone walls emerge as exemplifying optimal performance in terms of subjective restorative assessment, attaining the highest score of 1.13. Following closely are the red brick walls, which similarly manifest robust recovery attributes (score: 1.10). Conversely, the green plant and white mortar walls obtain relatively lower scores (0.34–0.35), significantly deviating from the other three wall types (Table 5, *p* < 0.01). This discrepancy implies their comparatively diminished subjective favorability. Notably, the preference for warm-toned street walls, as opposed to cool or neutral tones, becomes evident, correlating with a stress-reduction effect. This finding aligns with the findings of Costa et al.^54^.

**Table 5:** ANOVA analysis of RCS scores for different street walls.

Street wall type	Yellow mortar	Brown	Street wall type	Yellow mortar	Brown
Yellow mortar	1				
Brown stone	0.303	1			
Red brick	0.367	0.897	1		
Green plant	0.001**	0.000***	0.000***	1	
White mortar	0.000***	0.000***	0.000***	0.948	1

*p* < 0.001, ***highly significant; *p* < 0.01, **significant; *p* < 0.05, *weakly significant.

**Figure 5:**
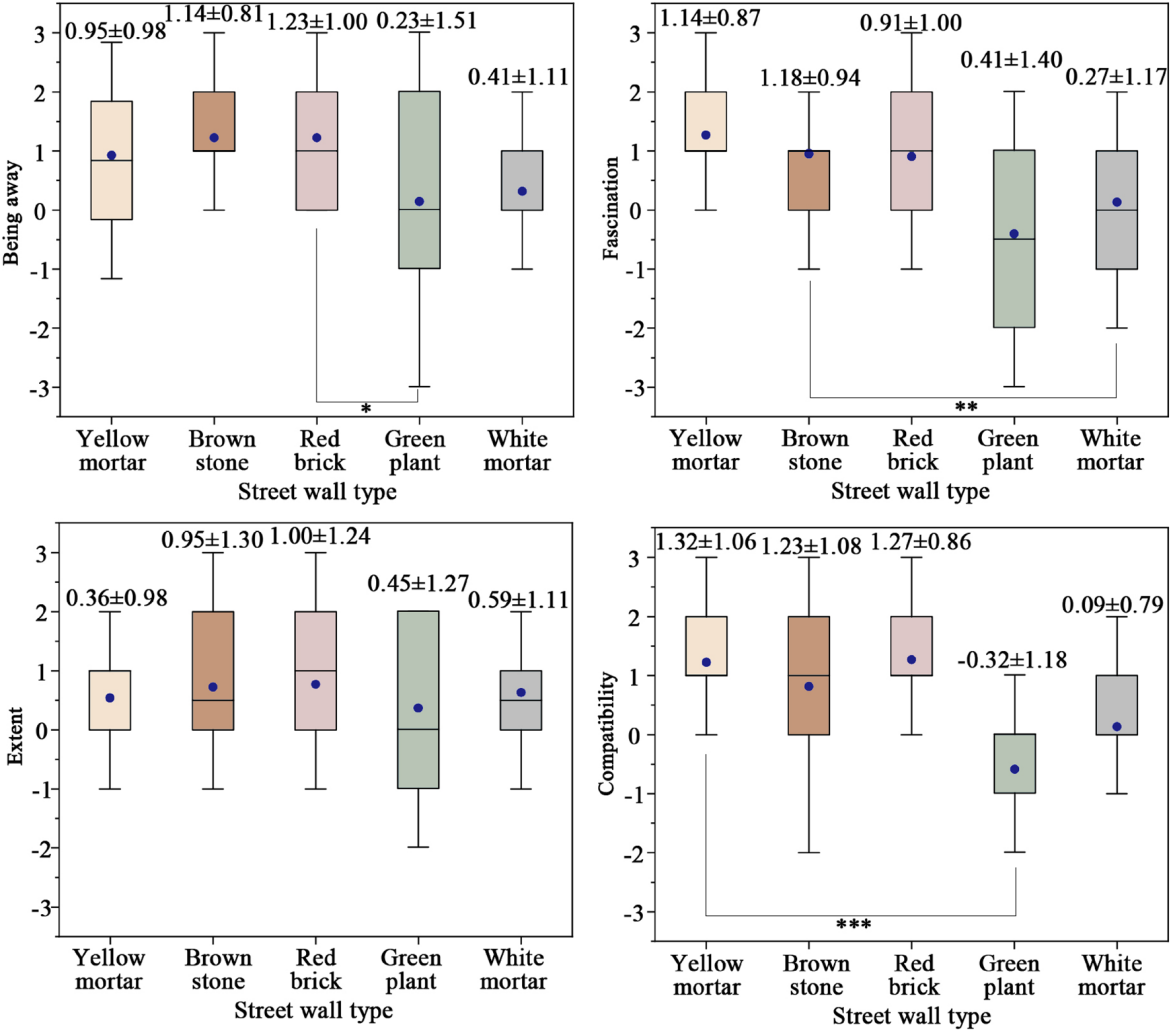
RCS scores for different street walls across four dimensions of ART.

Figure 5 shows RCS scores for different street walls across four dimensions of ART. Notably, walls adorned with brown stone, red brick, and yellow mortar achieved superior ratings in comparison to those covered with green plants and white mortar. For being away, warm-toned walls earned scores 0.54–0.82 higher than cool or neutral counterparts. Particularly, red brick walls secured the highest score (1.23). For fascination, the brown stone barrier attained the pinnacle score (1.18), reflecting heightened allure. While natural elements bolstered street appeal, walls cloaked with vegetation fared inadequately in this aspect, garnering a score of 0.41. In terms of extent, red brick and brown stone walls triggered a perceptible shift in attention, lowering stress levels, with ratings ranging from 0.95 to 1.00. As for compatibility, walls finished with yellow mortar netted the highest score (1.32), indicative of enhanced spatial integration and augmented mobility desire. Subsequently, brown stone and red brick walls followed with similar scores (1.23–1.27). Notably, warm-toned street walls outscored their counterparts by 1.13–1.64. Table 6 presents ANOVA results of RCS scores across four dimensions of ART, highlighting significant disparities between warm-toned and cool/neutral-toned walls. Street walls composed of brown stone, red brick, and yellow mortar exceeded those covered in white mortar and green plants across being away, fascination, extent, and compatibility. Overall, warm-toned street walls exerted a more pronounced positive influence on stress recovery.

**Table 6:** ANOVA analysis of RCS scores for different street walls across four dimensions of ART.

ART	Street wall type	Yellow mortar	Brown stone	Red brick	Green plant	White mortar
Being away	Yellow mortar	1				
	Brown stone	0.595	1			
	Red brick	0.426	0.791	1		
	Green plant	0.035*	0.009**	0.004**	1	
	White mortar	0.113	0.035*	0.018*	0.595	1
Fascination	Yellow mortar	1				
	Brown stone	0.893	1			
	Red brick	0.502	0.421	1		
	Green plant	0.033*	0.024*	0.141	1	
	White mortar	0.012*	0.008**	0.062	0.687	1
Extent	Yellow mortar	1				
	Brown stone	0.085	1			
	Red brick	0.175	0.901	1		
	Green plant	0.804	0.110	0.139	1	
	White mortar	0.536	0.323	0.266	0.710	1
Compatibility	Yellow mortar	1				
	Brown stone	0.771	1			
	Red brick	0.884	0.884	1		
	Green plant	0.002**	0.004**	0.003**	1	
	White mortar	0.000***	0.000***	0.000***	0.466	1

*p* < 0.001, ***highly significant; *p* < 0.01, **significant; *p* < 0.05, *weakly significant.

### Influence of different street walls on psychological responses

#### BP

**Figure 6:**
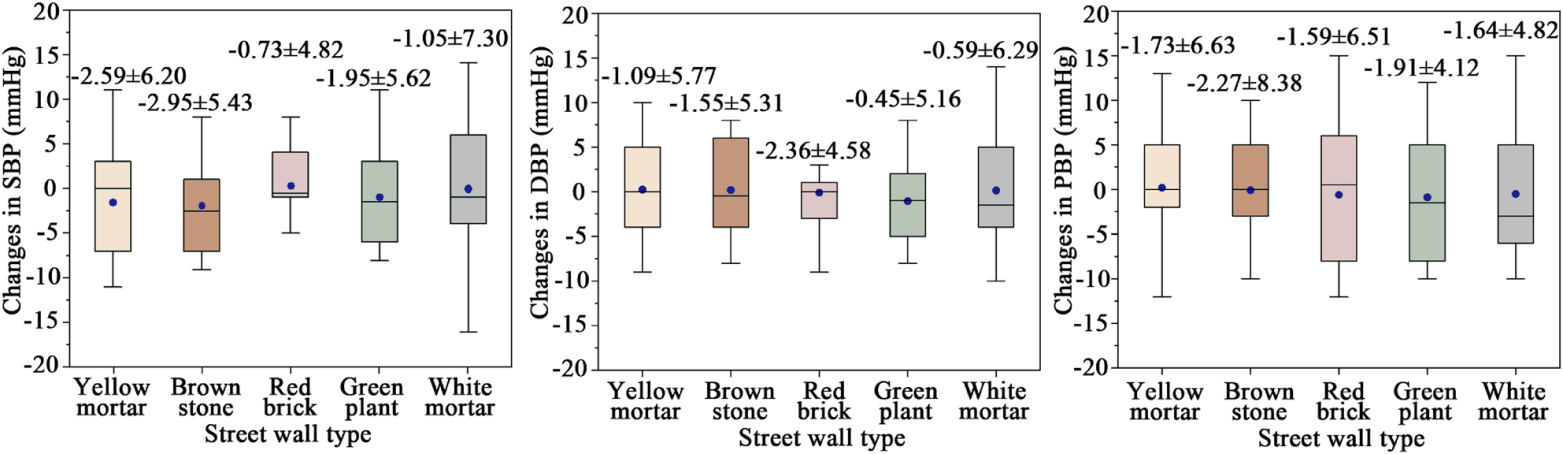
BP changes of different street walls.

Figure 6 shows BP changes of different street walls. Although no statistical difference was observed (*p* = 0.896), a reduction in both systolic and diastolic blood pressure was noted following exposure to the five different street wall simulations. For systolic blood pressure (SBP), brown stone walls exhibited the most pronounced recovery effect, achieving the greatest reduction (2.95 mmHg), followed by walls composed of yellow mortar, with a decrease of 2.59 mmHg. For diastolic blood pressure (DBP), the stress recovery trend ranked as red brick > brown stone > yellow mortar > white mortar > green plant. Notably, warm-toned walls presented superior decompression effects, boding well for cardiovascular health. Turning to pulse blood pressure (PBP), all five wall types exhibited stress-reducing effects. Of these, brown stone walls led with the most substantial decrease (2.27 mmHg), succeeded by green plant walls, showing a decrease of 1.91 mmHg. Overall, street walls featuring natural stone and greenery were associated with more favorable blood pressure outcomes.

#### HRV

**Figure 7:**
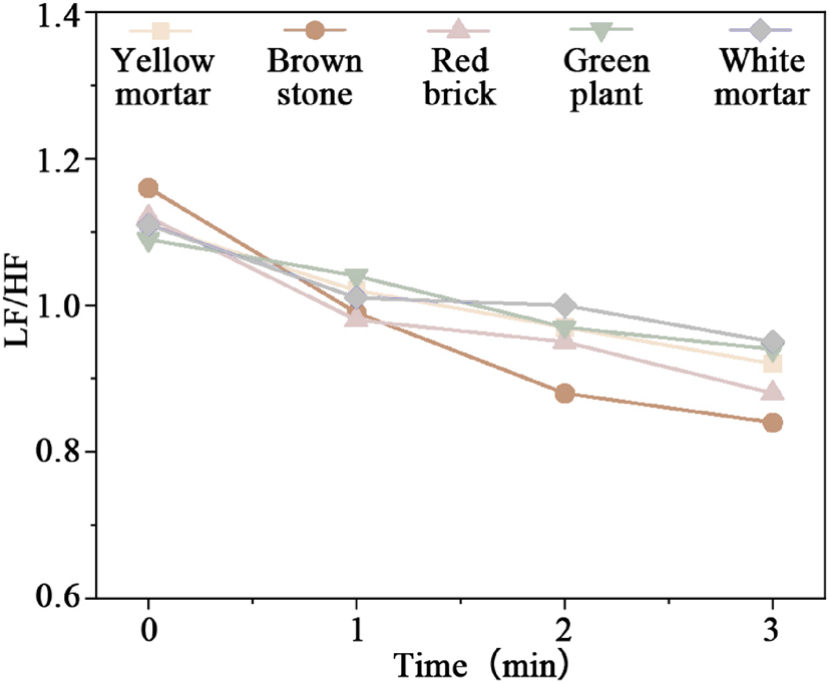
LF/HF changes of different street walls.

Figure 7 shows LF/HF changes of different street walls. Street walls featuring warm, cool, and neutral tones all contribute to stress recovery. The LF/HF experienced the swiftest and most pronounced decline (0.32, *p *< 0.05) in brown stone walls, facilitating quicker relaxation of both body and mind. Red brick walls followed with a reduction of 0.24. In general, warm-toned street walls were more effective in reducing the LF/HF and stress, supporting the findings of Park et al.^55^.

**Figure 8:**
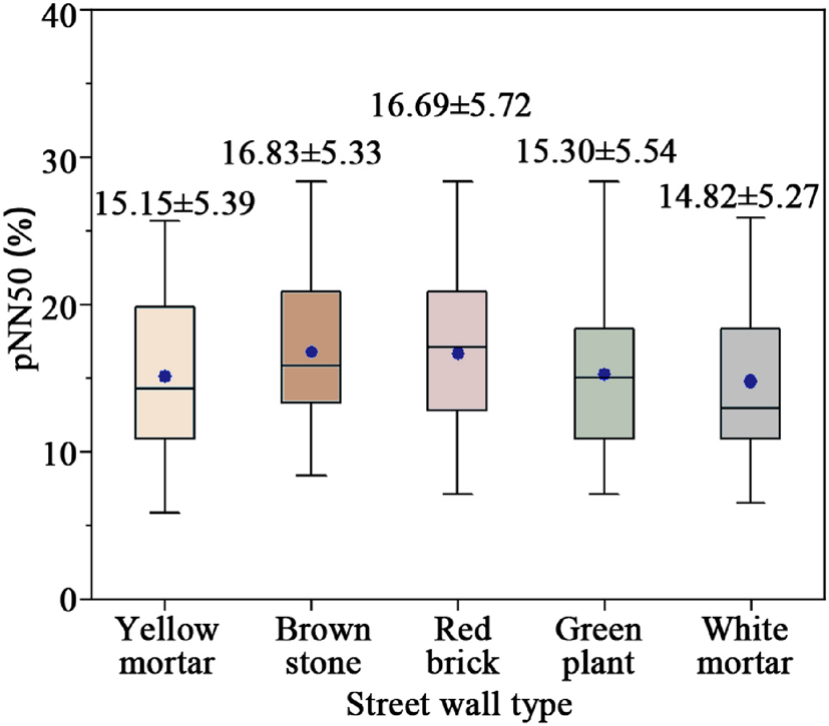
pNN50 changes of different street walls.

Figure 8 shows pNN50 changes of different street walls. Street walls crafted from brown stone and red brick exhibit superior performance in terms of pNN50, ranging from 16.69 to 16.83%. Following this, the ascending order of pNN50 efficacy encompasses street walls adorned with green plant, yellow mortar, and white mortar surfaces. Consistent with previous studies, walls that incorporate natural elements and enhance permeability demonstrate more effective restorative impacts on autonomic nervous system regulation^36,52^.

**Figure 9:**
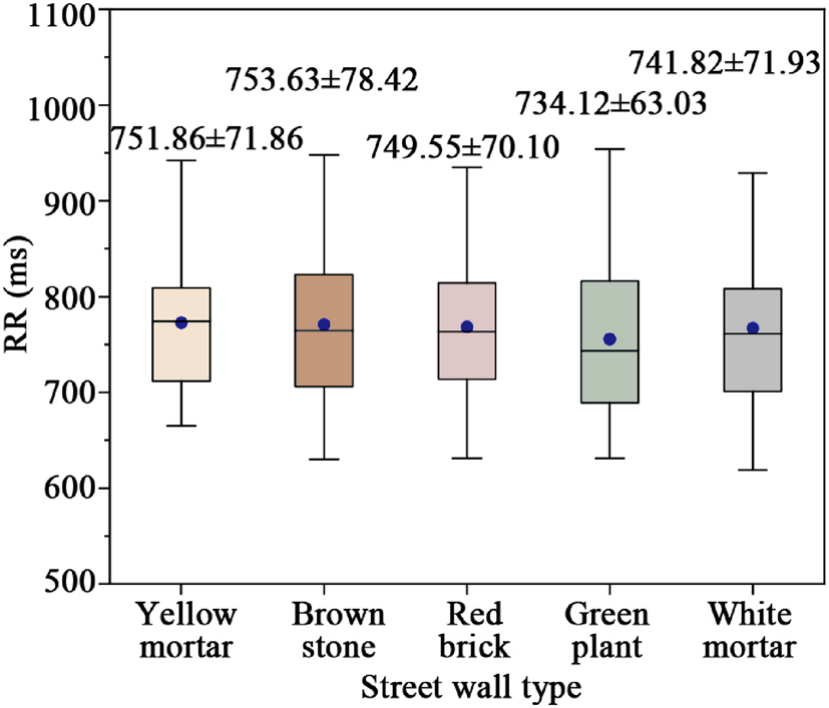
RR changes of different street walls.

Figure 9 shows RR changes of different street walls. Warm-toned street walls demonstrate elevated RR compared to cool and neutral tones, reflecting enhanced stress recovery efficacy. Notably, the brown stone wall boasts the highest RR (753.63 ms), surpassing street walls with yellow mortar, red brick, green plant, and white mortar by 1.57 ms, 4.08 ms, 19.51 ms, and 11.81 ms, respectively. Overall, warm-toned street walls positively influence cardiovascular activity and enhance stress recovery.

In summary, warm-toned walls, particularly brown stone walls, are effective in promoting physical and mental health by enhancing stress recovery levels in blood pressure and heart rate variability, thereby potentially reducing the risk of cardiovascular diseases^36,52^. Conversely, white mortar walls demonstrate a less significant effect on stress reduction.

### Pearson correlation analysis

Table 7 shows the Pearson correlation analysis of different street walls, encompassing both physical and psychological responses. Specifically, PBP, DBP, LF/HF, and pNN50 manifest correlations with restorative perception. PBP exhibits a negative correlation with the scores of RCS, being away, and fascination. Conversely, LF/HF presents a negative correlation with the scores of RCS and extent, while pNN50 demonstrates a positive correlation with the scores of compatibility. Notably, the congruence between physical and psychological responses outcomes mirrors a coherent trend in stress recovery. This alignment can be attributed to the interplay between physiological arousal and emotional responses, wherein restorative environments engender heightened positive emotions and a concomitant reduction in autonomic stress responses^56^.

**Table 7:** Pearson correlation analysis of different street walls.

r	Physiological responses				
	Being away	Fascination	Extent	Compatibility	RCS
Psychological responses					
LF/HF	− 0.101	− 0.125	− 0.219*	− 0.115	0.243*
pNN50	− 0.037	0.098	− 0.017	0.205*	0.106
RR	− 0.002	− 0.107	− 0.001	− 0.131	− 0.102
SBP	− 0.180	0.034	0.083	0.097	0.015
DBP	− 0.071	0.012	0.134	0.187	0.114
PBP	− 0.331**	− 0.397**	− 0.195	− 0.209*	− 0.486**

*p* < 0.001, *** highly significant; *p* < 0.01, ** significant; *p* < 0.05, * weakly significant.

## Discussion

This study indicates that warm colors not only foster a comfortable and pleasant spatial perception and maintain a positive mood but also enable individuals to feel relaxed and free from daily stress. These findings align with those of Costa et al.^54^. Furthermore, the combination of warm colors with surrounding greenery and architectural elements is favored by individuals, enhancing visual comfort and promoting physical and mental health in urban settings^57^. Additionally, the use of raw ecological materials like stone enhances the connection to nature and relaxation, supporting the pro-nature design theory that natural elements facilitate stress recovery and are preferred for their restorative qualities^55,57^. Feng et al. also advocate for the use of warm colors in designing color landscapes and incorporating natural elements to improve the stress recovery efficacy of physical environments, corroborating our experimental results^58^. Our findings further suggest that street walls built with red bricks, by increasing interface perspective, enhance sightline communication, broaden vision, spark exploratory interest, divert attention, and thus aid stress recovery^59^. Wang et al. also noted that an expanded visual perspective positively impacts stress recovery by fostering more pleasant emotions^60^.

Although greenery is typically associated with stress recovery, our study found limited performance in parameters other than blood pressure. This may be attributed to the potential oppressiveness of dense street trees, where a high rate of green vision can feel overwhelming^61^. Yeom et al. study supports this, suggesting that excessive plant density can reduce satisfaction, increase visual complexity, and induce negative psychological effects, such as claustrophobia and even biophobia, which hinder stress reduction^61^. As for white walls, their neutral color fails to induce significant emotional changes. The perception of color also depends on previous experiences^62^, potentially leading to feelings of boredom and monotony, and ultimately, a lesser restorative effect^63^.

In the context of urban renewal, we advocate for the preservation and promotion of street walls constructed from brown stone. These not only facilitate cultural heritage but also enhance stress recovery and improve mental and physical health. Particularly under the constraints of existing landscapes, incorporating natural elements to accentuate streetscape features and harmonizing landscape components—like utilizing blue and green spaces for activity areas in urban planning, and preserving historical contexts to augment positive urban renewal elements—can fulfill aesthetic and emotional public needs, thus fostering a healthier living environment. However, the generalizability of these findings requires further exploration. Although this study primarily utilized a VR environment, extending research to diverse cities and populations is crucial to validate the generalizability and applicability of the results. Field tests in varied real-world contexts will enable more precise evaluations of natural elements in urban renewal and allow for the adaptation of design strategies to different cultural and geographical settings.

To our knowledge, this is the first study that integrates VR, RCS, and physiological indicators to examine the impact of street walls' visual and textural features on stress recovery, aiming to enhance the humanization of urban spaces. Nonetheless, this study has limitations. While the sample size of 48 young college students may seem sufficient compared to other VR-based studies of built environments^64^, the selective nature and small size limit broad applicability. Participants were exclusively college students, providing a limited age group perspective; future research should include a more diverse demographic encompassing various genders, ages, and cultural backgrounds to assess aesthetic preferences comprehensively. Moreover, while five typical street walls in Qingdao were examined, the limited range of street scenes might lead to minimal observed differences. Future studies should incorporate a broader array of visual and textural features to deepen our understanding of their effects on stress recovery. Additionally, street environments are complex; factors such as street morphology, building facade composition, and traffic flow also influence stress recovery but were not thoroughly examined in this study and warrant further investigation.

## Conclusions

This study involved 48 participants to assess the effect of various historical street wall styles on stress recovery. By integrating psychological and physiological measures, the research aimed to identify street wall designs fostering residents' overall well-being, offering insights for human-centered urban street environment planning. The study's conclusions are summarized as follows:

- In psychological terms, warm-toned street walls are perceived as more restorative, as indicated by elevated Restorative Components Scale scores (0.94–1.13). Significantly, brown stone walls excel in assessing attributes such as being away, extent, and compatibility (0.95–1.23), exhibiting a stronger alignment with the characteristics of a restorative environment.
- Regarding BP, all five street walls exhibited a discernible reduction in stress. Notably, walls finished with brown stone, yellow mortar, and green plant demonstrated superior effects on diastolic and pulse blood pressures, registering reductions of 1.95–2.95 mmHg. Remarkably, the red brick wall yielded the most substantial decrease in systolic blood pressure (2.36 mmHg), potentially mitigating the risk of cardiovascular disease.
- Concerning heart rate variability, street walls made of brown stone demonstrated superior re-storability compared to other wall types. The low frequency/high frequency ratio exhibited the swiftest decrease, accompanied by pNN50 and RR elevated by 0.14–2.01% and 1.57–11.81 ms, respectively, when contrasted with other groups. These styles signify heightened activation of parasympathetic nerve activity, facilitating stress reduction.
- Both physically and psychologically, historical street walls in Qingdao consistently support residents' physical and mental recovery. Urban revitalization should prioritize warm-colored street walls, especially the brown stone masonry style.

## Data Availability

The data presented in this study are available on request from the corresponding author.

## Abbreviations

BP: Blood pressure
DBP: Diastolic blood pressure
HRV: Heart rate variability
ANS: Autonomic nervous system
PRS: Perceptual restorative scale
ANOVA: Analysis of variance
TSST: Trier social stress test
SBP: Systolic blood pressure
PBP: Pulse blood pressure
RCS: Restorative components scale
PNS: Parasympathetic nervous system
LF/HF: Low frequency/high frequency ratio
HMD: Head mounted display
ART: Attention recovery theory

## References

[1] Zhang N, Liu C, Hou C, Wang W, Yuan Q, Gao W (2024). The impact of indoor carbon dioxide exposure on human brain activity: A systematic review and meta-analysis based on studies utilizing electroencephalogram signals. Build. Environ..

[2] Bao X, Zhang T, Zeng Q, Dewancker BJ (2023). Adapting to changes in the COVID-19 pandemic: research and recommendations on spatial layout and resident experience in MURBs. City Built Environ..

[3] Jiang B, Li D, Larsen L, Sullivan WC (2014). A dose-response curve describing the relationship between urban tree cover density and self-reported stress recovery. Environ. Behav..

[4] Gaekwad JS, Sal Moslehian A, Roös PB (2023). A meta-analysis of physiological stress responses to natural environments: Biophilia and Stress Recovery Theory perspectives. J. Environ. Psychol..

[5] Kang J (2023). Soundscape in city and built environment: Current developments and design potentials. City Built Environ..

[6] Elsadek M, Liu B, Lian Z (2019). Green façades: Their contribution to stress recovery and well-being in high-density cities. Urban For. Urban Green..

[7] Finlay J, Franke T, McKay H, Sims-Gould J (2015). Therapeutic landscapes and wellbeing in later life: Impacts of blue and green spaces for older adults. Health Place.

[8] Bao X, Tao Z, Zeng Q, Dewancker BJ (2023). Adapting to changes in the COVID-19 pandemic: research and recommendations on spatial layout and resident experience in MURBs. City Built Environ..

[9] Korpilo S (2024). Landscape and soundscape quality promote stress recovery in nearby urban nature: A multisensory field experiment. Urban For. Urban Green..

[10] Aristizabal S (2021). Biophilic office design: Exploring the impact of a multisensory approach on human well-being. J. Environ. Psychol..

[11] Meng X (2024). Influence of air-conditioning intermittent operation on the cooling load from opaque envelopes in residences. Case Stud. Thermal Eng..

[12] Xu, F., Jin, A., Chen, X. & Li, G. New Data, Integrated Methods and Multiple Applications: A Review of Urban Studies based on Street View Images. 2021 IEEE International Geoscience and Remote Sensing Symposium IGARSS, 6532–6535, 10.1109/IGARSS47720.2021.9554660 (2021).

[13] Lin Y-H, Tsai C-C, Sullivan WC, Chang P-J, Chang C-Y (2014). Does awareness effect the restorative function and perception of street trees?. Front. Psychol..

[14] Van Dillen SM, de Vries S, Groenewegen PP, Spreeuwenberg P (2012). Greenspace in urban neighbourhoods and residents' health: Adding quality to quantity. J. Epidemiol. Commun. Health.

[15] Lohr VI, Pearson-Mims CH, Tarnai J, Dillman DA (2004). How urban residents rate and rank the benefits and problems associated with trees in cities. J. Arboricult..

[16] Lindal PJ, Hartig T (2015). Effects of urban street vegetation on judgments of restoration likelihood. Urban For. Urban Green..

[17] Lindal PJ, Hartig T (2013). Architectural variation, building height, and the restorative quality of urban residential streetscapes. J. Environ. Psychol..

[18] Bornioli A, Parkhurst G, Morgan PL (2018). Psychological wellbeing benefits of simulated exposure to five urban settings: An experimental study from the pedestrian's perspective. J. Transp. Health.

[19] Bornioli A, Parkhurst G, Morgan PL (2019). Affective experiences of built environments and the promotion of urban walking. Transp. Res. Part A Policy Pract..

[20] Asgarzadeh M, Koga T, Hirate K, Farvid M, Lusk A (2014). Investigating oppressiveness and spaciousness in relation to building, trees, sky and ground surface: A study in Tokyo. Landsc. Urban Plann..

[21] Cui F, Miyamori H, Kozaki M, Hirate K (2021). Research of the oppressiveness and spaciousness in urban space. J. Environ. Eng/.

[22] Demgenski P (2019). Dabaodao: The planning, development, and transformation of a Chinese (German) neighbourhood. Plann. Perspect..

[23] Demgenski, P. Seeking a Future for the Past: Negotiating Inner City Redevelopment and Heritage in Qingdao, China. (2015).

[24] Van den Berg AE, Jorgensen A, Wilson ER (2014). Evaluating restoration in urban green spaces: Does setting type make a difference?. Landsc. Urban Plann..

[25] Roe J, Aspinall P (2011). The restorative benefits of walking in urban and rural settings in adults with good and poor mental health. Health Place.

[26] Douglas IP (2022). Physical workplaces and human well-being: A mixed-methods study to quantify the effects of materials, windows, and representation on biobehavioral outcomes. Build. Environ..

[27] Jiaxing Z, Lin L, Hang L, Dongmei P (2021). Evaluation and analysis on suitability of human settlement environment in Qingdao. PLoS One.

[28] Li M, Pan J (2023). Assessment of influence mechanisms of built environment on street vitality using multisource spatial data: A case study in Qingdao China. Sustain. Basel.

[29] Lyu M (2022). Measuring the perceptual features of coastal streets: A case study in Qingdao, China. Environ. Res. Commun..

[30] Zhang N, Liu C, Li J, Hou K, Shi J, Gao W (2024). A comprehensive review of research on indoor cognitive performance using electroencephalogram technology. Build. Environ..

[31] Liu C, Zhang N, Wang Z, Pan X, Ren Y, Gao W (2024). Correlation between brain activity and comfort at different illuminances based on electroencephalogram signals during reading. Building and Environment.

[32] Kaplan S (1995). The restorative benefits of nature: Toward an integrative framework. J. Environ. Psychol..

[33] Laumann K, Gärling T, Stormark KM (2001). Rating scale measures of restorative components of environments. J. Environ. Psychol..

[34] Yin Y, Thwaites K, Shao Y (2022). Balancing street functionality and restorative benefit: Developing an expectation-current approach to street design. Sustain. Basel.

[35] Yu C-P, Lee H-Y, Luo X-Y (2018). The effect of virtual reality forest and urban environments on physiological and psychological responses. Urban For. Urban Green..

[36] Kabisch N, Püffel C, Masztalerz O, Hemmerling J, Kraemer R (2021). Physiological and psychological effects of visits to different urban green and street environments in older people: A field experiment in a dense inner-city area. Landscape Urban Plann..

[37] Park B-J (2007). Physiological effects of shinrin-yoku (taking in the atmosphere of the forest)—using salivary cortisol and cerebral activity as indicators. J. Physiol. Anthropol..

[38] Park B-J (2009). Physiological effects of forest recreation in a young conifer forest in Hinokage Town, Japan. Silva Fenn.

[39] de Brito JN (2020). The effect of green walking on heart rate variability: A pilot crossover study. Environ. Res..

[40] Zhang R-X, Zhang L-M (2021). Panoramic visual perception and identification of architectural cityscape elements in a virtual-reality environment. Fut. Gener. Comput. Syst..

[41] Xiang Y (2021). The comparisons of on-site and off-site applications in surveys on perception of and preference for urban green spaces: Which approach is more reliable?. Urban For. Urban Green..

[42] Guan H (2020). People's subjective and physiological responses to the combined thermal-acoustic environments. Build. Environ..

[43] Guan H (2020). Analysis of human electroencephalogram features in different indoor environments. Build. Environ..

[44] Liu C (2023). Preliminary data on effects of different street vegetation on stress recovery. Build. Simul..

[45] Jiang B, Chang C-Y, Sullivan WC (2014). A dose of nature: Tree cover, stress reduction, and gender differences. Landscape Urban Plann..

[46] Dickerson SS, Kemeny ME (2004). Acute stressors and cortisol responses: A theoretical integration and synthesis of laboratory research. Psychol. Bull..

[47] Benetos A, Thomas F, Bean KE, Pannier B, Guize L (2005). Role of modifiable risk factors in life expectancy in the elderly. J. Hypertens..

[48] Zhang B (2017). Reaction time and physiological signals for stress recognition. Biomed. Signal Process. Control.

[49] Jiang Y, Li N, Yongga A, Yan W (2022). Short-term effects of natural view and daylight from windows on thermal perception, health, and energy-saving potential. Building and Environment.

[50] Jin Z, Juan Y-K (2021). Is Fengshui a science or superstition? A new approach combining the physiological and psychological measurement of indoor environments. Build. Environ..

[51] Lei Q, Yuan C, Lau SSY (2021). A quantitative study for indoor workplace biophilic design to improve health and productivity performance. J. Clean. Prod..

[52] Castaldo R (2015). Acute mental stress assessment via short term HRV analysis in healthy adults: A systematic review with meta-analysis. Biomed. Signal Process. Control.

[53] Mukaka M (2012). Statistics corner: A guide to appropriate use of correlation in medical research. Malawi Med. J..

[54] Costa M, Frumento S, Nese M, Predieri I (2018). Interior color and psychological functioning in a university residence hall. Front. Psychol..

[55] Park SH, Lee PJ, Jung T, Swenson A (2020). Effects of the aural and visual experience on psycho-physiological recovery in urban and rural environments. Appl. Acoust..

[56] Ulrich RS (1991). Stress recovery during exposure to natural and urban environments. J. Environ. Psychol..

[57] Li KR, Yang YQ, Zheng ZQ (2019). Research on color harmony of building façades. Color Res. Appl..

[58] Miaomiao, F. Spatial renewal strategy of urban living Street under healingorientation: A case study of Dalian city, Dalian University of Technology, (2022).

[59] Hartsell AM (2021). Savanna hypothesis in the human–urban nature relationship. Open House Int..

[60] Wang R (2019). The relationship between visual enclosure for neighbourhood street walkability and elders’ mental health in China: Using street view images. J. Transp. Health.

[61] Yeom S, Kim H, Hong T (2021). Psychological and physiological effects of a green wall on occupants: A cross-over study in virtual reality. Build. Environ..

[62] Manav B (2017). Color-emotion associations, designing color schemes for urban environment-architectural settings. Color Res. Appl..

[63] Wang J, Zhang L, Gou A (2020). Study of the color characteristics of residential buildings in Shanghai. Color Res. Appl..

[64] Oselinsky K (2023). Virtual reality assessment of walking in a modifiable urban environment: a feasibility and acceptability study. Sci. Rep..

